# Spatial competition on the master-saliency map

**DOI:** 10.3389/fpsyg.2013.00394

**Published:** 2013-07-02

**Authors:** Ursula Schade, Cristina Meinecke

**Affiliations:** Department of Psychology and Sport Science, Institute of Psychology, University Erlangen-NurembergErlangen, Germany

**Keywords:** early vision, saliency map, master map, texture segmentation, retinal eccentricity, critical distance, non-classical receptive fields

## Abstract

The saliency map model (Itti and Koch, 2000) is a hierarchically structured computational model, simulating visual saliency processing. Iso-feature processing on feature maps and conspicuity maps precedes cross-dimensional signal processing on the master map, where the most salient location of the visual field is selected. This texture segmentation study focuses on a possible spatial structure on the master map. In four experiments the spatial distance between a texture irregularity in the stimulus (“target”) and a cross-dimensional task irrelevant texture irregularity in the backward mask (“patch”) was varied. The results show that the target-patch distance modulates target detection, and that this modulation is limited to critical distances around the target. We conclude that the signals from different feature dimensions compete on a spatial master map. There is first evidence that the critical distances increase with target eccentricity.

## Introduction

The saliency map model by Itti and Koch (2000), (Itti et al., 1998; adapted from Koch and Ullman, 1985) is a computational model in the context of artificial vision. It identifies visually salient locations by simulating human signal competition processes. The saliency map model consists of several hierarchically organized, two-dimensional processing levels (“maps”). At the feature map and conspicuity map levels basic-feature contrasts are calculated and weighted within each feature dimension separately (e.g., orientation, color, luminance). At the highest level within this model, which we refer to as the *master map* in the following, the signals from the subordinate maps are aggregated linearly. All feature information gets lost, whereas the spatial information is retained. A “winner take all” (WTA) mechanism scans over the whole master map and selects the most salient location in the visual field. Attention gets attracted first to this most salient location (Itti and Koch, 2000).

In the present study we aim to investigate the spatial structure on the master map. Itti and Koch (2000) proposed that signals compete by lateral inhibition processes within non-classical receptive fields at feature- and/or conspicuity level. According to the descriptions of the saliency map model by Itti and Koch (2000), however, we find no indication for spatial processing on the master map. We assume, however, that also on the master map spatial signal competition can occur, perhaps within spatial units analogously to receptive fields on the visual cortex. In the following, we refer to “spatial units” with respect to the computational saliency map model by Itti and Koch (2000), and to “receptive fields” when we relate to signal processing on the visual cortex.

There is already experimental evidence for spatial signal competition on feature map level from two texture segmentation studies (Schade and Meinecke, 2009, 2011). In those studies, a texture irregularity in the stimulus was the target to be detected, and a task-irrelevant texture irregularity (“patch”) was inserted into the backward mask; the spatial distance between target and patch was varied systematically. A distance-effect was observed; target detection was modulated by the target-patch distance. In Schade and Meinecke (2011) critical distances were found around the target, within which the patch impaired detection. Those critical distances probably indicate signal competition within spatially limited units on the feature maps, perhaps similarly to non-classical receptive fields (Itti and Koch, 2000). The critical distances increased with target eccentricity. Schade and Meinecke (2011) interpreted these eccentricity-dependent effects as possible crowding effects. In crowding experiments, usually a target letter is to be identified. When one (or multiple) task-irrelevant letter(s) appear(s) within a critical spatial distance around the target letter, letter identification gets worse. In crowding experiments, critical distances are typically larger when the target letter appears more peripherally than when it appears more centrally (e.g., Bouma, 1970; Andriessen and Bouma, 1976). Eccentricity-dependent critical distances, or Bouma's law, are considered strong evidence for crowding (e.g., Petrov and Popple, 2007; Pelli and Tillman, 2008). Crowding effects, however, are not found consistently in detection tasks (for a review of the crowding research see Levi, 2008). It has been assumed that the eccentricity-dependency of crowding may reflect the fact that receptive fields get larger in the periphery (Levi, 2008).

Also in visual search experiments spatial competition between two (or more) visual objects has been observed. For example, a distance effect is reported from two “additional singleton” studies, where a cross-dimensional distractor (e.g., color singleton) could appear additionally to the target (e.g., form singleton). Performance in orientation identification and orientation discrimination tasks varied also as a function of target-distractor distance (Mounts, 2000; Theeuwes and Chen, 2005). In a visual search study by Theeuwes et al. (2004) orientation discrimination was poorer with the distractor present than absent, but only when the distractor appeared close to the target. A more distant distractor had no effect on performance. Thus, regarding discrimination tasks, cross-dimensional signals seem to interact within critical distances. We assume that those critical distances indicate cross-dimensional signal competition within receptive fields on the visual cortex.

**Figure 1:**
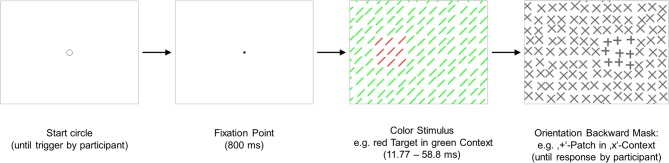
**Experiment 1: a graphical illustration of the sequence and timing of stimulus and backward mask on each trial**. Stimulus texture: with color-defined target. Mask texture: with irrelevant orientation-defined patch. The number of elements has been reduced and luminance contrast has been increased for better visualization of the texture structure.

The findings regarding target *detection*, however, are not so consistent. In a visual search study by Zehetleitner et al. (2009) reaction times slowed as a function of target-distractor distance, when a luminance-defined distractor appeared near to an orientation-defined target, but see Mounts (2000) for opposite findings. Processes of visual search and texture segmentation, however, should not be considered as identical (Wolfe, 1992; see also Meinecke and Donk, 2002; Schubö et al., 2004). Therefore, the present study aims to deliver evidence for a spatial structure on the master map, now in a texture segmentation paradigm. In texture segmentation studies interactions between two cross-dimensional texture gradients have already been observed (Callaghan, 1984; Callaghan et al., 1986; Koene and Zhaoping, 2007; Saarela and Landy, 2012). Until now, however, it is unknown, whether such interactions are dependent upon target-distractor distance. Our present study investigates the spatial structure on the master map by a paradigm analogous to that used by Schade and Meinecke (2011). To be sure that signal interactions occur on the master map, we use two *cross-dimensional* texture irregularities. A target in the stimulus is to be detected and a task-irrelevant cross-dimensional irregularity (“patch”) in the backward mask is to be ignored. In order to allow target and patch to overlap without changing the target elements, the patch is inserted into the texture of the backward mask (cf. Figure 1). The spatial distance between target and patch is varied systematically. If signals are processed in a spatial manner on the master map, the spatial distance between cross-dimensional signals should modulate detection performance, similar to that observed by Schade and Meinecke (2011) at feature map level. Critical distances, especially if they are eccentricity-dependent, would support the idea that it is crowding. Such interactions between two cross-dimensional visual objects within critical distances would indicate signal competition within spatial units on the master map.

In Experiments 1 and 2, the target and the task-irrelevant patch are defined by color and orientation dimensions (and vice versa); in Experiment 3, the target is an orientation-defined contrast, and the task-irrelevant patch is a luminance contrast. Experiment 4 aims to test whether the distance effect persists when target and patch appear without temporal delay. To this end an orientation-defined target and a luminance-defined patch now are both inserted into the stimulus, and the target-patch distance is varied.

We expect target detection performance to vary in a distance-dependent manner, with detection improving with increasing target-patch separation up to a critical separation. In Experiment 3 the target can appear at ±3.9 or at ±9.2°. We expect larger critical distances around the more peripheral than around the more central target, analogously to the findings by Schade and Meinecke (2011), who found increasing critical distances with increasing retinal eccentricity of the target.

## Experiment 1: color-defined target, orientation-defined patch

The effects of an irrelevant orientation contrast on the detection of a color-defined target were tested. The target was a color contrast. An orientation contrast in the mask was the patch that should be ignored (cf. Figure 1). For explorative reasons target color was varied in two conditions. In the Red Target condition target elements were red, and context elements were green; in the Green Target condition target elements were green and context elements were red. The colors red and green were physically iso-luminant. This experiment explored whether the detection of a color-defined target is weaker with the orientation-defined patch present than absent, and whether any such performance impairment is distance-dependent. Finally, we investigated whether critical distances exist around the target, by varying spatial distance between target and patch systematically between 0 (target and patch overlapping) and 15.04°.

### Methods

#### Participants

Six students, five female and one male, were paid or received course credit for participating in this experiment. Ages were 18–22 years; mean age was 20.5 years (*SD* = 1.5). All participants had normal or corrected to normal visual acuity and normal red-green vision. All experiments described in this manuscript were undertaken with the understanding and written consent of each subject.

#### Apparatus

The experiment was run on an iMac 3.06 GHz Intel Core 2 Duo with Mac OSX 10.6.2. Stimuli were presented on an Iiyama HM 704 UTC monitor at 85 Hz (39.6 × 30.2°, 1024 × 768 pixels), and stimulus presentation was controlled by a MATLAB program using the Psychophysics Toolbox (Brainard, 1997; Pelli, 1997). Luminance was measured with a Minolta luminance meter (model LS-110). Visual acuity was tested by a Rodenstock R22 vision tester, stimulus no. 212. Participants sat at a table on which a head- and chinrest was mounted. Viewing distance was 47 cm, with the direction of gaze inclined slightly downward. Participants responded to the stimuli by pressing one of two mouse buttons with the index finger of either hand.

#### Stimuli

The stimuli (see Figure 1) consisted of a 67 × 51 element array, subtending a visual angle of 38.7 × 29.5°. The elements were 45° tilted lines of 11 pixels length. The distance between adjacent line elements was 15 pixels horizontally and vertically. The target was composed of a square 3 × 3 array of elements. Whereas the vertical position of the target was held constant in the middle of the stimulus (determined by the position of its central element), its horizontal position was varied. The target could appear at ±3.5°. A jitter randomly displaced the position of each texture element by 0, 1, 2, or 3 pixels (in the horizontal and/or vertical direction).

The mask consisted of spatially overlapping orthogonal elements each forming an *x*-like figure (see Figure 1). Twenty-one mask versions contained a patch, which consisted out of 3 × 3 elements (1.7 × 1.7°) analogous to the target in the stimulus. The elements of the patch were ±like figures (cf. Figure 1). One mask version contained no patch. Patch position varied on 21 eccentricities along the horizontal meridian (0°, ±1.2°, ±2.3°, ±3.5°, ±4.6°, ±5.8°, ±6.9°, ±8.1°, ±9.3°, ±10.4°, ±11.6°). Thus, 14 different spatial distances between target and patch were possible (0°, 1.2°, 2.3°, 3.5°, 4.6°, 5.8°, 6.9°, 8.1°, 9.3°, 10.4°, 11.6°, 12.7°, 13.9°, 15.0°). All other mask parameters were as those of the stimulus. The context lines in the stimulus texture were green (48 cd/m^2^), and the target lines in the stimulus were red (48 cd/m^2^). In an inverse condition the target lines were green, and the context lines were red. The backgrounds of the textures were gray (103 cd/m^2^). The elements in the mask were dark-gray (59 cd/m^2^).

#### Procedure

Three sessions were administered on 3 different days, each session lasting approximately 50 min. The first session was for training; data from this session were not analyzed further. In this session the Red Target and Green Target condition was practiced in alternating blocks. Each experimental session started with two practice blocks of 88 trials (50% trials containing a target). In each of the two experimental sessions the Red Target condition or the Green Target condition was implemented. The sequence of conditions was permutated over participants. A backward mask followed the stimulus in all trials. Each of the 22 mask versions (21 versions with patch, one homogeneous version) appeared four times. Each possible combination of patch position and target position appeared two times in each block. All trials were presented in random order. Each trial started with a small circle (diameter of 11 pixels) displayed at the center of the screen informing the participant that he or she could start the next trial by simultaneously pressing both mouse buttons. The circle was replaced by a fixation point (2 × 2 pixels) and after 800 ms the stimulus followed. In order to avoid ceiling or floor effects presentation times of the stimuli (SOAs between stimulus and mask) were adapted to each participant's individual detection skills and ranged from 11.77 to 25.43 ms (*M* = 21.57 ms, *SD* = 4.80 ms) in the Red Target condition, and from 35.30 to 58.8 ms in the Green Target condition (*M* = 45.10 ms, *SD* = 11.57). The SOA was kept constant for each person throughout each condition. The mask remained on the screen until the participant responded by pressing either the left button (no target present) or the right button (target present). A short, single acoustic click informed the participant that his or her response was correct; a short double click that she or he had made an error. Then the circle was displayed on the screen again, indicating that a new trial could be initiated. The participants were told that this study investigates the human visual system. They were shown the stimulus with the target and the backward mask with the task-irrelevant patch. The possible positions of the target and of the patch on the screen were also indicated. Participants were requested to maintain central eye fixation and to respond quickly. They were enjoined to answer only with “yes,” if they are sure to have seen a target and to press always the “no” button when they were not sure. Thus, the false alarm rate should be kept down to a minimum. The purpose of this instruction was to keep individual differences in criterion as low as possible. Since the hit rate varied as a function of the spatial distance between the patch and the target, participants could define their criterion only in relation to negative trials (trials without a target). As proposed by Treisman and Watts (1966; see also, Neyman and Pearson, 1933), in such an experimental situation in which the signal strength varies within the experimental condition, it makes sense to use only the false alarm rate as instruction for the participants to set their criterion. Independent variables were the target-patch distance (degree of visual angle). Dependent variables were hits and false alarms. Reaction times were measured in order to identify and exclude outlier trials from the statistical analysis.

### Results

For each participant, all trials in which reaction time exceeded the mean for that block by three standard deviations were dropped from further analyses. For statistical analysis, data were averaged over the trials of the respective condition. For comparison of means *t*-tests for paired samples (two tailed) and ANOVAs for repeated measures were calculated, if not commented otherwise. When possible, sensitivity (*d*') was calculated. For target-patch distances, *d*'-values could not be calculated since the false alarms in no-target trials could not be attributed unambiguously to the (absent) left *or* right target. For example, when the patch appeared on −6.9°, a false alarm could pertain to the target at +3.5° (distance: 9.4°) or to the target at −3.5° (distance: 3.5°). Effect sizes are reported (partial η^2^, η^2^_*p*_) for *F*-tests. In all figures standard error bars were calculated for within subject designs, according to the suggestions by Cousineau (2005) and Morey (2008), for illustration (not for statistical analysis).

A Two-Way ANOVA on “Target Color” (red, green) and on “Patch Presence” (absent, present) with sensitivity (*d*') as dependent measure reveals a significant effect of “Patch Presence” [*F*_(1, 5)_ = 46.14, *p* < 0.01, η^2^ = 0.90], no effect of “Target Color” and no interaction “Target Color” × “Patch Presence,” indicating that the patch impaired target detection in both Target Color conditions [“Red Target”: *d'(patch absent)* = 3.18, *d'(patch present)* = 1.97; “Green Target”: *d'(patch absent)* = 3.73, *d'(patch present)* = 1.97]. A Two-Way ANOVA was calculated on the factors “Color” (2 levels, red, green) and “Patch position” (21 positions), revealing a significant effect of “Patch position” [*F*_(20, 100)_ = 3.10, *p* < 0.001], but no effect of “Target Color” [*F*_(1, 5)_ = 1.50, *p* = 0.28, n.s.], and no interaction [*F*_(20, 100)_ = 0.71, *p* = 0.81, n.s.]; thus the data of the two color conditions were averaged for further analysis.

**Figure 2:**
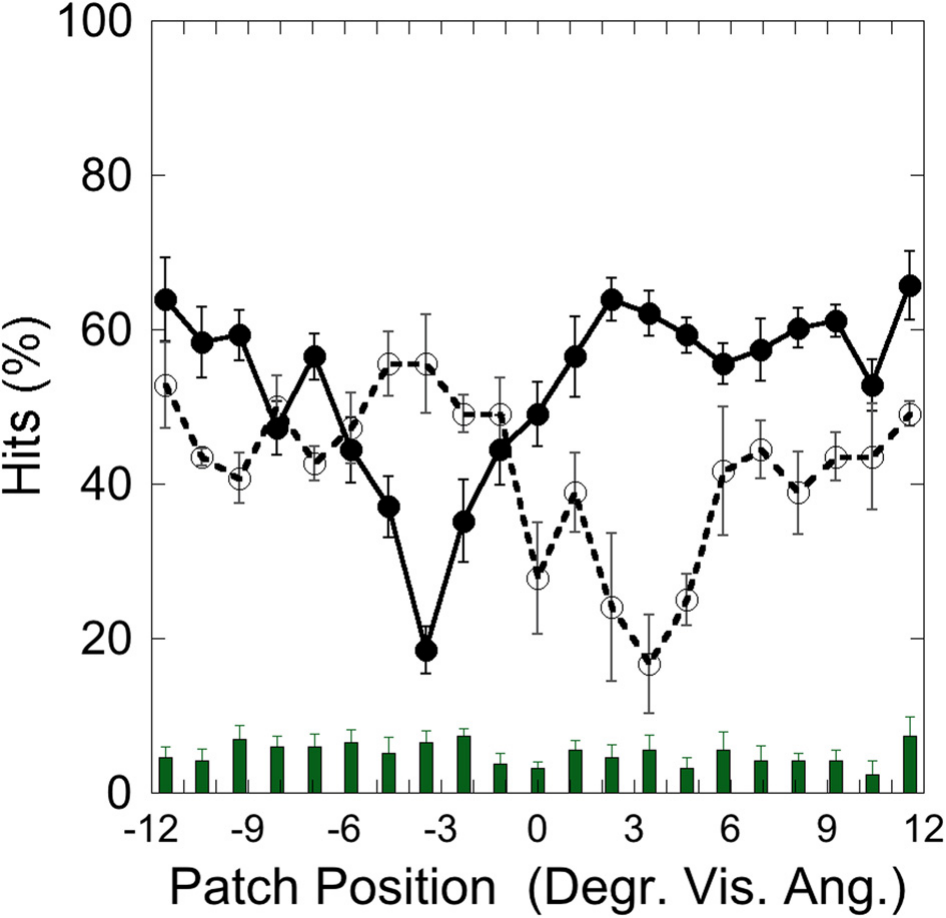
**Experiment 1: hits and false alarms averaged over color conditions as a function of patch position**. Solid line and filled circles show hits on the target at −3.5°, dotted line and unfilled circles show hits on the target at +3.5°. The columns on the bottom depict false alarms as a function of patch position. Black bars depict standard errors.

Figure 2 depicts the hits on the left (−3.5°) and on the right (+3.5°) target and false alarms, as a function of patch position, illustrating that performance was weakest with patch and target overlapping and increased with increasing patch-target distance.

**Figure 3:**
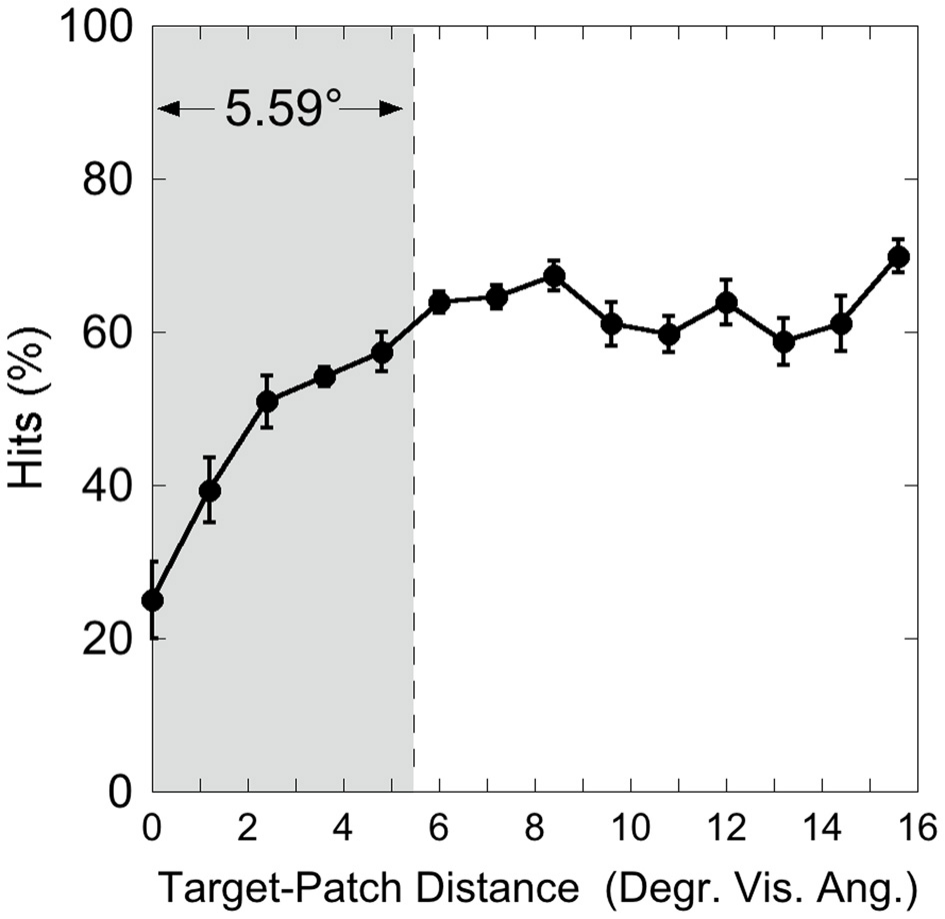
**Experiment 1: hits as a function of target-patch distance, averaged over the Red Target and Green Target condition**. The gray field represents the critical distance (averaged over participants). Black bars depict standard errors.

Figure 3 shows the hit rate as a function of distance, suggesting a distance-effect by the patch. A One-Way ANOVA was calculated on the factor “Target-Patch Distance” (14 levels: distances from 0° up to 15.6°), with hits the dependent measure. This analysis revealed a significant effect of “Distance” [*F*_(13, 65)_ = 15.39, *p* < 0.001, η^2^ = 0.76], indicating that performance varied as a function of distance between target and patch. In order to find out whether the patch produces critical distances around the target, an inverted exponential fit derived from Equation (1)

**Figure 4:**
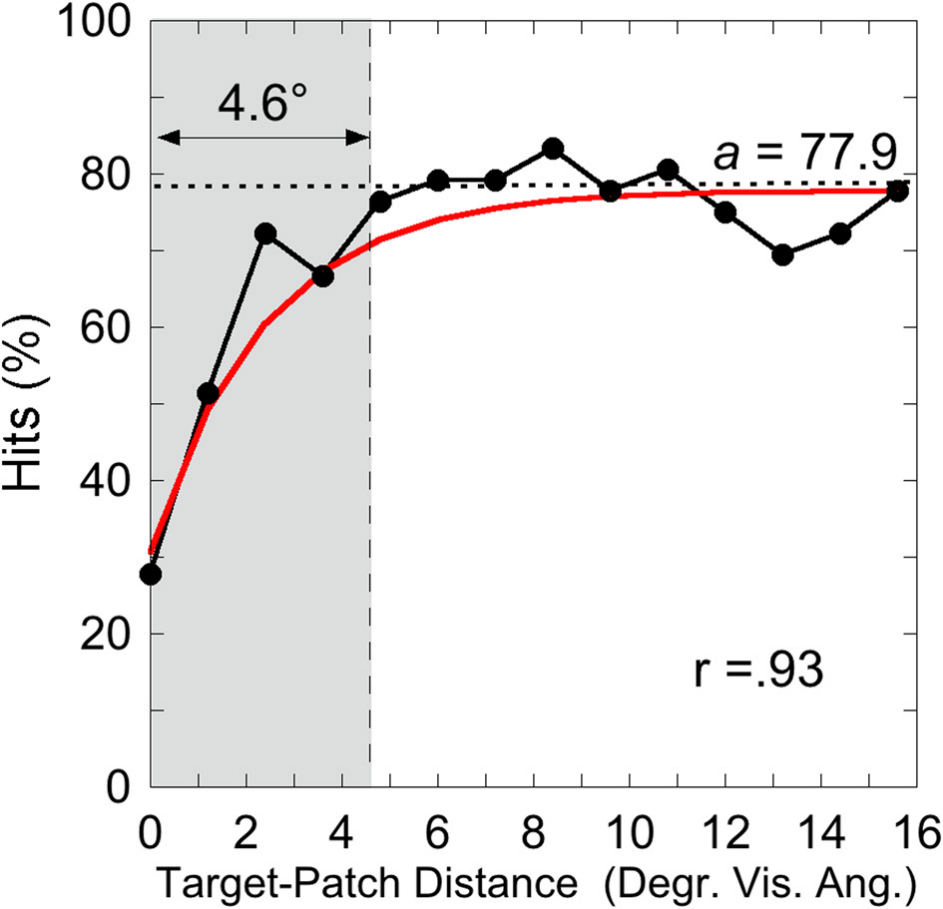
**Experiment 1: hits by Participant SL as a function of target-patch distance converging to the value *a* (dotted line)**. The gray field represents the critical distance (90% from *a*). The inverse exponential function fits the data well (*r* = 0.93).

(1) y=a×[1−exp(−x/b)]

was applied on individual hit rates as a function of target-patch distance, according to the method applied by Schade and Meinecke (2011). The *y*-axis-value (*y*) equals the inverted exponential function with diffraction factor (*b*) and *x*-axis-value (*x*) with asymptotic shift factor (*a*). The individual critical distance was defined as the distance, where the *y*-values reach 90% of the *a*-value. This method is similar to that applied by Yeshurun and Rashal (2010) in determining critical distances. Figure 4 exemplarily depicts the hits of participant SL as a function of target-patch distance, the fitting and the critical distance (gray field). This procedure was applied to all participants. The averaged critical distance is 5.59° (cf. Figure 3) with a standard deviation of *SD* = 2.68°. The model fits the hit functions well (mean *r* = 0.76, Fisher's *Z* transformed).

In sum, the signals from the orientation-defined patch interacted with the signals from the color-defined target. Detection was impaired in the presence of the patch and varied with the spatial distance between target and patch. An inverted exponential function fitted the data well, indicating that the orientation-defined masking patch impaired the detection of the color-defined target within a critical distance around the target.

## Experiment 2: orientation-defined target, color-defined patch

**Figure 5:**
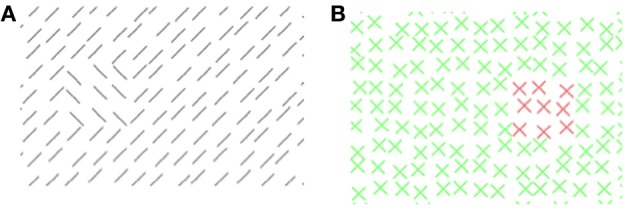
**Experiment 2: (A) stimulus texture with orientation-defined target. (B)** Mask texture with color-defined patch. **(A,B)** The number and luminance of elements has been reduced for better visualization of the texture structure.

In Experiment 1, we found that within a critical distance detection of color-defined targets was impaired by the presence of a task-irrelevant orientation-defined patch in the backward mask. In the second experiment we investigated whether the inverse is also true (cf. Figure 5). Again, patch color was varied in two conditions. In the Green Patch condition the patch was a green among red elements, in the Red Patch condition the patch was red among green elements. It was tested whether the color-defined patch impairs the detection of the orientation-defined target, whether this impairment is distance-dependent, and whether critical distances exist.

### Methods

#### Participants

Six female students were paid or received course credit for participating in this experiment. Ages were 20–32 years, and mean age was 22.3 years (*SD* = 2.1).

#### Apparatus

The apparatus was the same as in Experiment 1.

#### Stimuli

The stimuli consisted of a 59 × 45 element array, subtending a visual angle of 38.7 × 29.5°. The elements were 45° tilted lines of 13 pixels length. The distance between adjacent line elements was 17 pixels horizontally and vertically. The target (2 × 2°) was made up of 3 × 3 elements whose orientation was orthogonal to that of context elements. The target could appear at ±3.9°. As in Experiment 1, jitter randomly displaced the position of each texture element by 0, 1, 2, or 3 pixels (in horizontal and/or vertical direction). The elements of the mask consisted of *x*-like figures, as in Experiment 1, the context elements were green, and the patch elements were red. In the inverse condition the patch elements were green and the context elements were red. Nineteen mask versions contained a patch, which consisted out of 3 × 3 elements (2 × 2°), corresponding to the size of the orientation-defined target. One mask version contained no patch. Patch position varied on 19 eccentricities along the horizontal meridian (0°, ±1.3°, ±2.6°, ±3.9°, ±5.2°, ±6.6°, ±7.9°, ±9.2°, ±10.5°, ±11.8°). Thus, 13 different spatial distances between target and patch were possible (0°, 1.3°, 2.6°, 3.9°, 5.2°, 6.6°, 7.9°, 9.2°, 10.5°, 11.8°, 13.1°, 14.4°, 15.7°). All other mask parameters were as those of the stimulus. Luminance values were: backgrounds were gray (80 cd/m^2^); the oriented context and target lines in the stimulus texture were dark gray (21 cd/m^2^). The green and red elements in the mask both had a luminance of 43 cd/m^2^.

#### Procedure

The procedure was similar to that in Experiment 1, but in this experiment each experimental block contained 160 trials, and six blocks were performed in each condition. SOAs ranged from 35.3 to 70.6 ms in both conditions (Red Patch: *M* = 49.2, *SD* = 13.8 ms; Green Patch: *M* = 50.1 ms, *SD* = 12.2 ms).

### Results

A Two-Way ANOVA on “Patch Color” (red, green) and on “Patch Presence” (absent, present) with sensitivity (*d*') as dependent measure reveals a significant effect of “Patch Presence” [*F*_(1, 5)_ = 22.94, *p* < 0.01, η^2^ = 0.82], no effect of “Patch Color” and no interaction “Patch Color” × “Patch Presence,” indicating that the color-defined patch in the mask impairs the detection of the orientation-defined target equally in both patch color conditions [“Red Patch”: *d'(patch absent)* = 3.65, *d'(patch present)* = 2.10; “Green Patch”: *d'(patch absent)* = 2.86, *d'(patch present)* = 1.59]. A further Two-Way ANOVA on the factors “Patch Color” (red, green) and “Patch Position” (19 positions) with hits the dependent measure revealed a significant effect of “Patch Position” [*F*_(18, 90)_ = 4.66, *p* < 0.001], no effects of “Patch Color” [*F*_(1, 5)_ = 2.26, *p* = 0.19, n.s.] and no interaction between “Patch Position” × “Patch Color” [*F*_(18, 90)_ = 0.78, *p* = 0.72, n.s.]. Therefore, data were averaged over the color conditions for further analysis.

**Figure 6:**
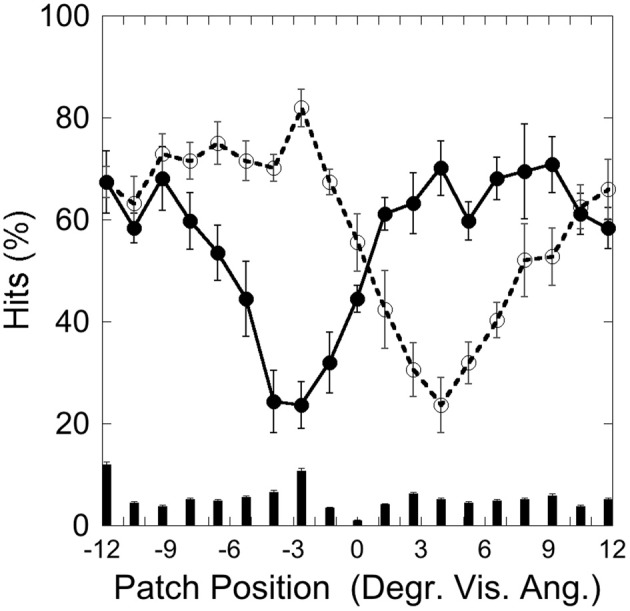
**Experiment 2: hits and false alarms averaged over patch color conditions as a function of patch position**. Solid line, filled circles show hits on the target at −3.9°, dotted line, unfilled circles show hits on the target at +3.9°. The columns on the bottom depict false alarms as a function of patch position. Black bars depict standard errors.

Figure 6 depicts the hits on the left (−3.9°) and right (+3.9°) target and false alarms, as a function of patch position. It shows that detection is weakest with patch and target overlapping and that detection increases with increasing target-patch distance, as in Experiment 1.

**Figure 7:**
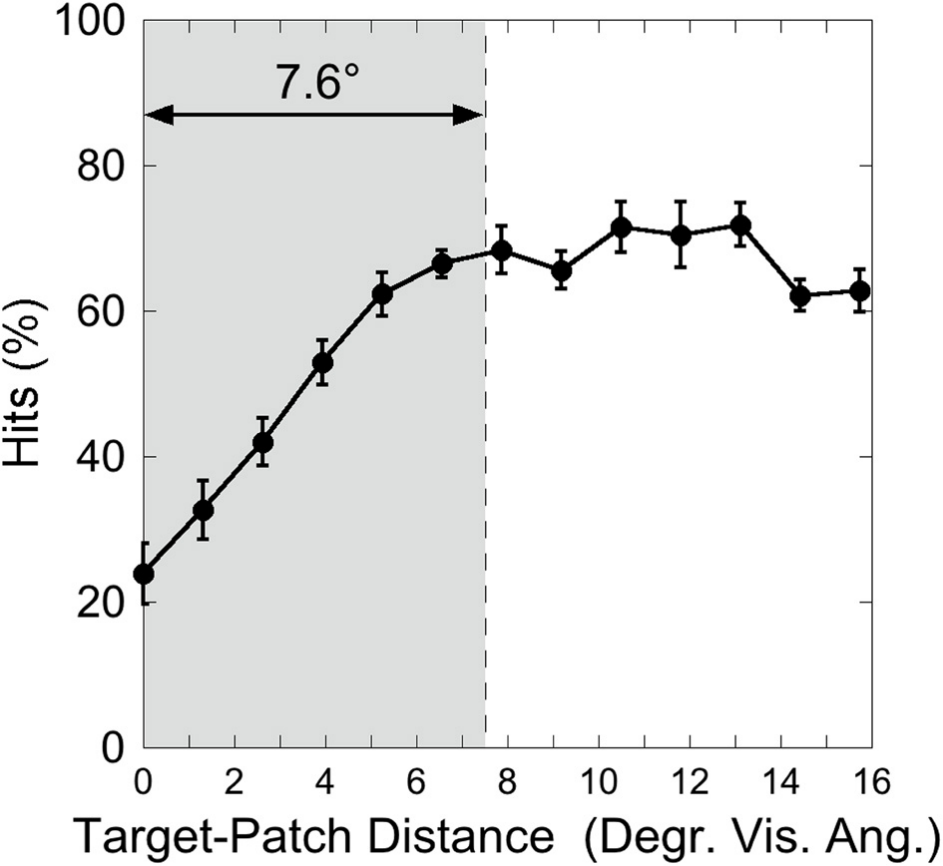
**Experiment 2: hits as a function of target-patch distance, averaged over the conditions “Red Patch” and “Green Patch.”** The gray field represents the critical distance (averaged over participants). Black bars depict standard errors.

Figure 7 depicts the hit rate (averaged over participants) as a function of the distance between target and patch. As in Experiment 1, a One-Way ANOVA was calculated on the factor “Target-Patch Distance” (13 levels: distances from 0 up to 16°), revealing a significant effect of “Target-Patch Distance” [*F*_(12, 60)_ = 21.1, *p* < 0.001, η^2^ = 0.81]. In order to test whether the color-defined patch produces critical distances around the orientation-defined target, an inverted exponential fit derived from Equation 1 was applied, as in Experiment 1. The critical distance, averaged over participants was 7.6° (*SD* = 2.9°), as the gray field in Figure 7 shows. The inverse exponential function fits the data well (mean *r* = 0.76, Fisher's *Z* transformed).

Thus, in Experiment 2, as in Experiment 1, the saliency signals from the color patch interacted with the saliency signals from the orientation-defined target. The deleterious effect of the patch in the mask on target detection was modulated by the spatial distance between target and patch. The inverted exponential function fits the data well, so that critical distances could be determined.

## Experiment 3: orientation-defined target, luminance-defined patch, two target eccentricities

**Figure 8:**
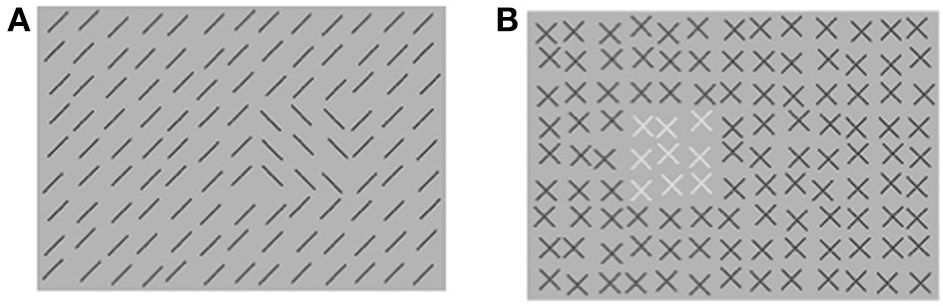
**Experiment 3: (A) stimulus texture with orientation-defined target. (B)** Mask texture with luminance-defined patch. **(A,B)** The number of elements has been reduced for better visualization of the texture structure. For the same purpose the luminance-defined contrast in panel **(B)** has been increased compared to the original mask.

In order to further examine the generalizability of the results of Experiment 1 and 2 we now used an orientation-defined target and a luminance-defined patch (cf. Figure 8). The spatial distance between target and patch was varied systematically as in Experiments 1 and 2. Additionally, target-eccentricity was varied in two conditions. In the Central Target condition the target could appear at ±3.9°, as in Experiment 2, and in the Peripheral Target condition the target could appear at ±9.2°. As in Experiments 1 and 2 it was investigated whether the irrelevant patch impairs target detection in a distance-dependent manner and whether critical distances exist. We tested additionally, whether the critical distance increases with target eccentricity, perhaps similar to the findings regarding iso-feature effects in the orientation dimension (Schade and Meinecke, 2011).

### Methods

#### Participants

Six female and two male students were paid or received course credit for participating in this experiment. Ages were 20–23 years, and mean age was 21.4 years.

#### Apparatus

The apparatus was the same as in Experiments 1 and 2.

#### Stimuli

Stimulus elements were the same as in Experiment 2, with the following exceptions: all target and context elements in the stimulus as well as the context elements in the mask were black (6.4 cd/m^2^). Patch elements in the mask were white (87.6 cd/m^2^). Backgrounds in stimulus and mask were gray (80.2 cd/m^2^). In the Central Target condition, the target could appear at −3.9° or +3.9°, as in Experiment 2. Note that the target is presented at an eccentricity of ±3.9°, *not* in the fovea. In the Peripheral Target condition the target could appear at −9.2° or +9.2°. As in Experiment 2, the patch could appear on one of 19 eccentricities along the horizontal meridian (0°, ±1.3°, ±2.6°, ±3.9°, ±5.2°, ±6.6°, ±7.9°, ±9.2°, ±10.5°, ±11.8°). In the Central Target condition 13 different spatial distances between target and patch were possible, as in Experiment 2 (0°, 1.3°, 2.6°, 3.9°, 5.2°, 6.6°, 7.9°, 9.2°, 10.5°, 11.8°, 13.1°, 14.4°, 15.7°). In the Peripheral Target condition 17 different distances were possible (0°, 1.3°, 2.6°, 3.9°, 5.2°, 6.6°, 7.9°, 9.2°, 10.5°, 11.8°, 13.1°, 14.4°, 15.7°, 17.0°, 18.4°, 19.7°, 21.0°).

#### Procedure

The procedure was similar to that in Experiment 2, but now 5 sessions on 5 different days were administered; one training session, and two experimental sessions per condition. SOAs ranged from 23.53 to 47.06 ms (*M* = 33.82 ms) in the Central Target condition, and from 23.53 to 35.30 ms in the Peripheral Target condition (*M* = 26.47 ms). Independent variables were target eccentricity (±3.9° in the Central Target condition, and ±9.2° in the Peripheral Target condition) and the target-patch distance. Dependent variables were hit rate and false alarm rate.

### Results

**Figure 9:**
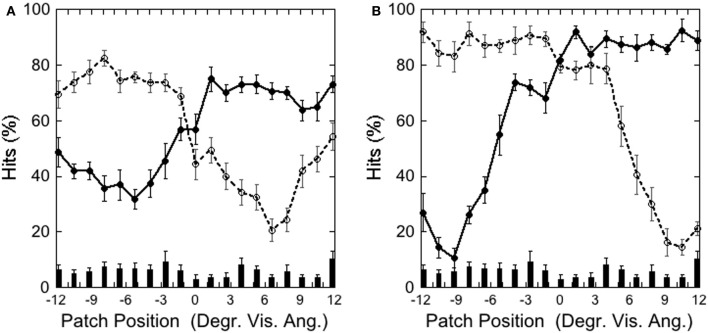
**Experiment 3: (A) Central Target (±3.9°) and (B) Peripheral Target (±9.2°): hits and false alarms as a function of patch position**. Solid lines and filled circles show hits on the left-side target, dotted line and unfilled circles show hits on the right-side target. The columns on the bottom depict false alarms as a function of patch position. Black bars depict standard errors.

Figure 9 depicts the hits on the left and right target as a function of patch position in the **(A)** Central Target and **(B)** Peripheral Target condition, illustrating that performance was weakest with the patch and target overlapping, and increased with increasing patch-target distance, as in Experiments 1 and 2.

In order to determine whether the patch impaired target detection a Two-Way ANOVA was calculated on the factor Target Eccentricity (two levels: “Central Target,” “Peripheral Target”) and Patch (two levels: Present, Absent), as in Experiments 1 and 2. This analysis reveals a significant main effect of Patch [“Central Target”: *d'(patch absent)* = 2.77, *d'(patch present)* = 2.30, “Peripheral Target”: *d'(patch absent)* = 2.44, *d'(patch present)* = 1.83; *F*_(1, 7)_ = 40.95, *p* <.001, η^2^ = 0.85], and no further effects nor interactions, indicating that the patch impaired detection in both conditions.

**Figure 10:**
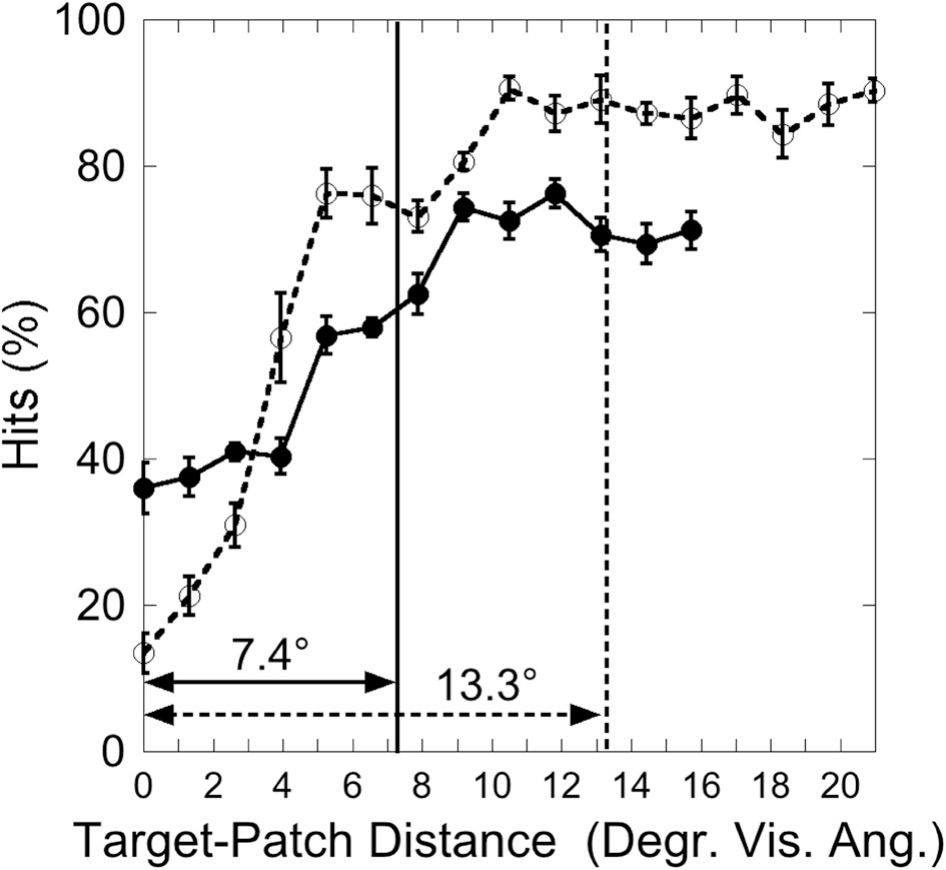
**Experiment 3: hits as a function of target-patch distance**. Vertical lines represent the critical distance (averaged over participants). Solid lines and filled circles: Central Target condition; dotted lines and unfilled circles: Peripheral Target condition. Black bars depict standard errors.

Figure 10 depicts the hits as a function of target-patch distance in the two target eccentricity conditions. We tested if the target-patch distance modulates detection. Note that in the Peripheral Target condition 17 different target-patch distances were applied, in the Central Target condition, however, only 13 distances were possible. Therefore, we calculated a Two-Way ANOVA on the factors “Target-Eccentricity” (two levels: central, peripheral) and “Distance” with only 13 levels on the factor “Distance.” There was a significant effect of “Distance” [*F*_(12, 84)_ = 93.77, *p* < 0.001, η^2^ = 0.93], no effect of “Target-Eccentricity” [*F*_(1, 7)_ = 0.95, *p* = 0.36, η^2^ = 0.12], and a significant “Target-Eccentricity” × “Distance” interaction [*F*_(12, 84)_ = 16.41, *p* < 0.001, η^2^ = 0.70]. This indicates that detection varied as a function of distance, but differently in the two conditions. Two One-Way ANOVAs were calculated separately for each condition on the factor “Distance” (“Central Target” 13 levels, “Peripheral Target” 17 levels), revealing a larger effect (η^2^) on “Distance” in the Peripheral Target condition (η^2^ = 0.91) than in the Central Target condition (η^2^ = 0.84).

It was now investigated whether the impairing patch effect was restricted to a critical distance around the target. As in Experiments 1 and 2 an inverted exponential function was fitted on individual hit functions, and the critical distance was defined as the target-patch distance where the function reaches 90% from the parameter *a*. In this experiment two participants were excluded from this analysis because their data did not reach the *a*-value in the Central Target condition, i.e., the estimated critical distance was exceptionally large. This exclusion criterion is similar to that applied by Yeshurun and Rashal (2010). The inverse exponential function fits the data well in both conditions (“Central Target”: *mean R* = 0.85; “Peripheral Target”: *mean R* = 0.92), indicating critical distances around the target. As depicted in Figure 10, the critical distance is larger in the Peripheral Target condition (13.3°) than in the Central Target condition [7.4°; *T*_(5)_ = 2.07, *p* < 0.05, one-sided].

In sum, as in Experiments 1 and 2, the saliency signals interacted in a distance-dependent manner. Critical distances were observable. In this experiment the distance effect was larger, and the critical distance was larger when the target appeared more peripherally.

## Experiment 4. orientation-defined target, luminance-defined patch, both in stimulus

**Figure 11:**
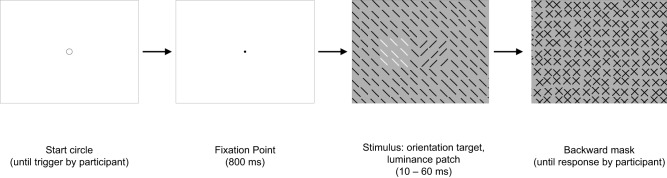
**Experiment 4: a graphical illustration of the sequence and timing of stimulus and backward mask on each trial**. Stimulus texture: 50% with orientation-defined target and 100% with irrelevant luminance-defined patch. Mask texture with no irregularities. The number of elements has been reduced and luminance contrast has been increased for better visualization of the texture structure. In this example the target-patch distance is small.

In contrast to Experiments 1–3, in this experiment, the task-irrelevant patch was not inserted into the mask, but into the stimulus together with the target. Thus, target and patch appeared exactly at the same time (cf. Figure 11). The patch could be either in the vicinity of the target (center to center distance of 2.3°) or further away (center to center distance of 8.1°). The purpose of this experiment was to test whether the target-patch distance modulates detection also, when there is no temporal delay between target and patch presentation.

### Method

#### Participants

Six female students were paid or received course credit for participating in this experiment. Ages were 19–20 years, and mean age was 20.0, years.

#### Apparatus

The apparatus was the same as in Experiments 1–3.

#### Stimuli

Form and structure of stimulus and mask were identical to that of Experiment 1, with the following exceptions: stimulus and mask backgrounds were gray (60.8 cd/m^2^). Stimulus and mask elements were dark-gray (21 cd/m^2^), with exception of the irrelevant patch. The patch now consisted of white elements (89.6 cd/m^2^). The patch was now inserted into the stimulus texture. The mask texture contained now no irregularity (cf. Figure 11).

#### Procedure

The procedure was similar to that in Experiment 1, with the following exceptions: in this experiment only one session comprising about 50 min was administered. Eight training blocks (à 64 trials) and five blocks (à 128 trials) were performed. Only eight different stimulus versions, containing a patch were applied (cf. Table 1). SOAs ranged from 50 to 60 ms (*M* = 54). The independent variable was the target-patch distance, and the dependent variable was the hit rate.

**Table 1:** **Experiment 4: possible positions of target and patch**.

**Target position**	**Patch position**	**Target-patch distance(°)**
−3.5°	−11.6°	8.1
	−5.8	2.3
	−1.2	2.3
	+6.9	8.1
+3.5	−6.9	8.1
	+1.2	2.3
	+6.9	2.3
	+5.8	8.1

### Results

**Figure 12:**
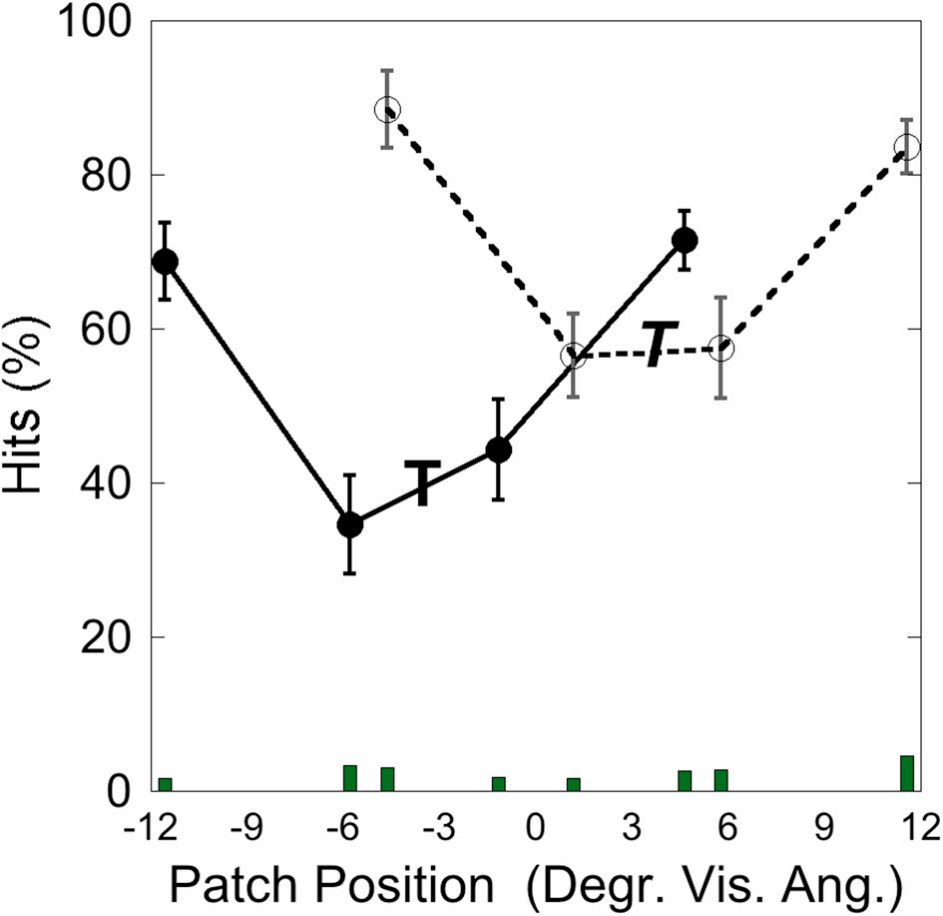
**Experiment 4: hits and false alarms as a function of patch position**. The “T” marks the position of the target at −3.5°, the italic “*T*” indicates the target position at +3.5°. Solid lines and filled circles show hits on the −3.5°, target, dotted line and unfilled circles show hits on the +3.5° target. The columns on the bottom depict false alarms as a function of patch position. Black bars depict standard errors.

Figure 12 depicts the hits as a function of patch position with respect to the target at −3.5° and at +3.5°. Performance was poorer when the patch appeared close to the target than when it appeared more distant from it, as in Experiment 1–3. A comparison of means showed that performance was significantly weaker when the target-patch distance was 2.3° (*M* = 48.25; *SD* = 17.01) than when this distance was 8.1° [*M* = 78.11; *SD* = 8.91; *t*_(4)_ = 4.27, *p* < 0.05], similar to the patterns found in Experiments 1–3.

## Discussion

### Summary of results

This study aimed to investigate the spatial structure on the saliency master map by Itti and Koch (2000; Itti et al., 1998). It was assumed that signals from lower order units representing distinct stimulus dimensions (e.g., color vs. orientation) compete within spatial units on the higher order master map. In four experiments a texture irregularity in the stimulus (target) was to be detected. A task-irrelevant cross-dimensional texture irregularity was inserted into the backward mask (patch). In an additional experiment (Experiment 4), the irrelevant patch was inserted into the same stimulus as the target and thus appeared simultaneously with the target. The spatial distance between target and patch was varied systematically. Expectedly, detection was weaker with the patch present than absent. The deleterious effect of the patch defined by a stimulus dimension distinct from that of the target was most pronounced with the patch close to the target and decreased with increasing distance. This effect persisted when target and patch appeared simultaneously. Beyond a critical distance around the target this distance effect was no longer observable, and target detection remained stable despite further increasing the target-patch distance. There is evidence from Experiment 3 that critical distances are larger around the more peripheral than around the more central target.

Our finding of *cross-dimensional interference* is in line with earlier findings in texture segmentation and in visual search. In texture segmentation studies investigating the co-processing of orientation- and color-irregularities, reaction times slowed and performance worsened when the irrelevant irregularity was in a different location to that of the target (Callaghan et al., 1986; Callaghan, 1989; Zhaoping and May, 2007; Saarela and Landy, 2012).

The *distance-dependency* of the patch-effect in our study is similar to that reported by Schade and Meinecke (2009, 2011), however, they studied *iso*-dimensional texture irregularities (orientation). Cross-dimensional distance effects and critical distances were observed in visual search tasks, as mentioned in the Introduction Section. As far as we know, however, until now only iso-dimensional distance-effects and critical distances have been investigated in a texture segmentation task (Schade and Meinecke, 2011).

The *larger critical distances* around the more peripheral than the more central target observed in Experiment 3 of our study are in line with the findings by Schade and Meinecke (2011) regarding iso-dimensional interactions and corresponds to the assumption by Meinecke (1989) that the size of processing units relevant for texture segmentation increases with retinal eccentricity. The evidence from our experiment is weak, however, because two participants have been excluded from the analysis of critical distances. Only further research can clarify whether critical distances between cross-dimensional signals increase in the periphery. Eccentricity-dependent critical distances would strongly support Bouma's law indicating crowding in such texture segmentation tasks.

### Implications for the saliency map model by Itti and Koch (2000)

The saliency map model (Itti et al., 1998, 2000; Itti and Koch, 2000) is structured hierarchically. Spatial signal interactions are assumed to occur only on the iso-dimensional feature and conspicuity map level, but not on the master map. On the master map the signals from the feature maps are linearly summed, and a Winner Takes All (WTA) mechanism selects the most salient location of the whole visual field. In our experiments signals from different feature maps interacted in a spatially dependent manner and within critical distances. In order to explain our data in terms of the saliency map model by Itti and Koch (2000), we would propose the introduction of an additional spatial competition mechanism on the master map, logically before the WTA mechanism selects the most salient location. We have shown that cross-dimensional interactions are strongly dependent upon the relative separation between target signal and target-irrelevant signals.

### Implications for the V1 saliency map model (e.g., Li, 2002)

Alternatively, our data can be explained by a more recent model of the saliency map on V1 by Zhaoping and coworkers (Li, 2000, 2002). Li Zhaoping and coworkers delivered evidence for the saliency map on V1 (e.g., Zhaoping and Dayan, 2006; Koene and Zhaoping, 2007; Zhaoping, 2008, 2012). The *V1 hypothesis* states that a WTA mechanism selects just the receptive field (RF) of the most efficient V1-cells for further attentional processing, while ignoring the feature tuning of the cell. Intra-cortical interactions in V1 cause “iso-feature suppression” (e.g., Gilbert and Wiesel, 1983; Allman et al., 1985) between nearby neurons that are tuned to similar features. These interactions allow inhibition of neurons, even when their receptive fields are not overlapping (e.g., Li, 2002). How can the V1 model explain spatially determined cross-dimensional interactions or critical distances? Koene and Zhaoping (2007) point out that not only does V1 contain cells tuned to one unique feature, but also conjunctive cells that are tuned to two features, e.g., for color *and* orientation (Hubel and Wiesel, 1959; Johnson et al., 2004). Koene and Zhaoping (2007) assume that the activity of conjunctive cells gets inhibitory input not only from other conjunctive cells tuned to the same feature combination, but also from single feature cells sensitive to either of the features. Recent experimental research indicates that texture and color are not processed independently (Saarela and Landy, 2012). Indeed, a recent electrophysiological single cell recording demonstrates that in macaque V1 exist color-processing cells that are sensitive to color and also to orientation (Johnson et al., 2008).

One difficulty to adapt our data to the concept of conjunctive cells, as proposed by Koene and Zhaoping (2007), is that in our experiments target and patch did not appear simultaneously; the patch appeared after the target. Experiment 4, however, shows that the distance effect was also observable when the patch appeared simultaneously with the target. We assume that signal processing in texture segmentation must comprise a certain time window. It seems that in our experiments the patch followed the target in such short time that signals were processed co-actively. However, more research is needed to investigate whether the distance effects and critical distances depend on certain temporal properties associated with backward masking between the various feature domains used in this study.

## Conclusion

In a texture segmentation task target detection was impaired by a cross-dimensional task-irrelevant texture irregularity. Impairment was modulated by the target-patch distance, under the condition that the patch appeared within a critical distance around the target. If we explain our data in terms of the saliency model by Itti and Koch (2000) we would postulate additional spatial competition on the master map. If we apply the V1 model by Zhaoping (e.g., Li, 2002), we would explain spatial effects and the critical distances in our study by signal competition in the receptive fields of conjunctive cells (Koene and Zhaoping, 2007), that are sensitive for color and orientation, and/or for luminance and orientation.

### Conflict of interest statement

The authors declare that the research was conducted in the absence of any commercial or financial relationships that could be construed as a potential conflict of interest.
